# A Multi-Stage Computer Vision Framework for Real-Time Fire Detection in Low-Light Multi-Camera Environments

**DOI:** 10.3390/s26165038

**Published:** 2026-08-08

**Authors:** Ruhi Taş

**Affiliations:** Department of Computer Engineering, OSTIM Technical University, Ankara 06374, Turkey; ruhi.tas@ostimteknik.edu.tr

**Keywords:** fire detection, smoke detection, YOLO11, multi-camera surveillance, night-vision enhancement, CLAHE, MOG2 background subtraction, region-of-interest filtering, false-alarm suppression, real-time video analytics, ablation study, FIgLib

## Abstract

**Highlights:**

**What are the main findings?**

- The detector is a YOLO11s model that attains a mean average precision (mAP@0.5) of 0.65 (F1 = 0.70) on a leakage-free held-out test set. A five-stage post-detection pipeline—illumination enhancement, neural detection, motion validation, ROI exclusion, and temporal hysteresis—filters candidate detections before an alarm is raised; in a leave-one-out ablation on non-fire video, motion validation is the dominant false-alarm suppressor, reducing a raw-detector baseline of eight false alarms to zero.
- On external video, the end-to-end system reaches an event-level recall of 44–61% depending on the operating point, with a median time to first alarm of about one to three seconds. The system runs at approximately 10-20 FPS, sustains multiple concurrent streams, and inter-annotator agreement is substantial (Cohen’s κ = 0.75).

**What are the implications of the main findings?**

- Pipeline-level engineering (motion cues, spatial filtering, temporal reasoning) yields larger operational gains than swapping one YOLO variant for another. Each stage targets a distinct failure mode.
- The system shows that on commodity Windows hardware, a dedicated GPU is helpful but not strictly required.

**Abstract:**

This paper presents NexFire Pro, a deployable desktop framework for real-time fire and smoke surveillance across multiple simultaneous IP or USB camera streams. The research contribution is a failure-mode-driven pipeline design methodology: each of the five post-detection stages is derived from a distinct, identifiable failure-mode category (illumination drift, static false-positive objects, spatially expected heat sources, temporal noise), evaluated with a leakage-free, source-disjoint held-out protocol that avoids the temporal data leakage that frame-level splits introduce. A fine-tuned YOLO11s backbone serves as the fixed detection substrate, surrounded by a five-stage processing pipeline: (1) CLAHE and adaptive gamma correction; (2) neural detection; (3) MOG2-based motion validation; (4) normalised-coordinate ROI exclusion; and (5) temporal alarm hysteresis. On a leakage-free held-out image test set, the detector attains mAP@0.5 = 0.65 (F1 = 0.70). A leave-one-out ablation on non-fire video shows that motion validation is the dominant false-alarm suppressor, reducing a raw-detector baseline of eight false alarms to zero. The proposed design-and-evaluation framework is intended to be transferable to other reliability-oriented computer vision applications beyond fire detection.

## 1. Introduction

Fire incidents cause significant loss of life and economic damage across residential, industrial, and public environments. Data from the National Fire Protection Association show that tens of thousands of fires ignite annually in U.S. industrial and manufacturing facilities [1]. Because fire spreads rapidly in its early stages, detecting it within the first seconds of ignition often determines whether suppression is possible.

Conventional detection relies on point sensors—ionisation detectors, heat sensors, and multi-criteria units—which are cheap, widely installed, and limited to a small sensing volume. Dust, steam, or sudden temperature shifts often trigger false alarms, and operator trust erodes accordingly [2,3]. Video-based detection has emerged as a complementary approach, exploiting existing surveillance infrastructure to monitor wide areas [2,4].

Single-stage YOLO-family detectors are now the workhorse of applied work because they balance accuracy against inference speed [5,6,7].

Illumination variability is the first such problem. When cameras switch to near-infrared (IR) mode at night, models trained on visible-spectrum imagery lose much of their spectral discrimination. Classical preprocessing—CLAHE and gamma correction—closes part of this gap at negligible cost [8,9]. Static objects that resemble fire—indicator lights, reflective surfaces, furnace glows—are a persistent source of false positives. Adding motion information is a long-standing remedy [10,11,12]. Industrial scenes often contain expected hot regions, so an ROI exclusion mechanism is operationally necessary [13]. Single-frame detections are noisy on their own. Temporal consistency filtering removes transient lighting artefacts [14,15]. Combining these mechanisms—motion validation, ROI filtering, and temporal hysteresis—is well established in the surveillance literature [15,16]. What is less well established is how the components interact and how much of the operational improvement comes from each.

The answer, as the ablation study in Section 5.2 demonstrates under a leakage-free, source-disjoint held-out protocol, is both substantial and component-specific: each stage suppresses a distinct, identifiable failure mode.

Rather than proposing a new detector architecture (recent work in this direction includes SmokeyNet [17], EFSD-YOLO [18], DSS-YOLO [6], and Yar et al. [7]), the present work addresses a different and largely open research question: given a fixed backbone, which post-detection filtering stages produce what magnitude of reliability gain, through which mechanism, and to what degree do they interact? Each pipeline stage is designed around a specific, identifiable failure mode category—illumination drift, static false-positive objects, spatially expected heat sources, and temporal noise—and the leave-one-out ablation protocol isolates each stage’s contribution under a held-out evaluation that prevents the temporal data leakage common in frame-level splits. This failure-mode-driven design methodology, together with the leakage-free, source-disjoint held-out protocol, constitutes the primary research contribution of this paper.

(i) A failure-mode-driven pipeline design methodology in which each of the five stages—illumination-aware preprocessing, neural detection, motion validation, normalised-coordinate ROI exclusion, and temporal alarm hysteresis—is derived from and mapped to a distinct, identifiable failure mode category observed in real industrial deployments, rather than assembled ad hoc.
(ii) A leakage-free, source-disjoint held-out evaluation protocol—in which the test set shares no source with the training data and every duplicate or near-duplicate frame is removed so that no test image (or a near-duplicate of it) appears in training—together with a leave-one-out ablation study that quantifies the false-alarm reduction attributable to each stage independently, providing reproducible evidence of component-level contributions rather than end-to-end system performance alone.
(iii) A deployable multi-stream implementation that applies the same pipeline concurrently to heterogeneous sources (IP/USB cameras, Internet streams, and pre-recorded video) at approximately 120 FPS single stream on commodity hardware; controlled multi-stream throughput profiling is identified as future work.
(iv) An automatic IR/night-mode preprocessing stage (inter-channel-deviation mode detection with CLAHE enhancement) for low-light operation, addressing a regime that is rarely documented in the fire-detection literature; a quantitative labelled near-infrared evaluation is identified as future work.
(v) Public release of the training configuration, data-split manifest, and evaluation scripts; trained model weights will be made available upon journal acceptance via a persistent repository, enabling independent replication of all reported results.
(vi) A reusable, reliability-oriented design methodology—bringing together failure-mode analysis, leakage-free evaluation, and per-stage quantification—that is not specific to fire detection and can be generalised to other safety-critical computer vision applications.

The remainder of this paper is structured as follows. Section 2 surveys related work. Section 3 describes the system architecture. Section 4 details the principal algorithmic components. Section 5 reports experimental results. Section 6 and Section 7 present the discussion and concluding remarks.

## 2. Related Work

### 2.1. Deep Learning-Based Fire and Smoke Detection

Early vision-based approaches relied on hand-crafted features: colour models, motion cues, and wavelet-based texture descriptors [14,19,20,21]. These were interpretable but generalised poorly across environments. Deep convolutional classifiers then narrowed the gap on standard benchmarks [8,22,23], and the field moved to data-driven representations. Single-stage detectors dominate applied work today [24,25]. Fire-YOLOv5 [9,26] reports a mean average precision of 90.5% at near-real-time throughput. YOLOv8 variants aimed at smart-factory scenarios reach comparable figures [27]. Recent architectures push further through attention and feature fusion: EFSD-YOLO builds on YOLOv11 [18], and DSS-YOLO is a lightweight YOLOv8 variant [6,28,29]. For wildland smoke, SmokeyNet [17] couples a per-tile ResNet-34 feature extractor with an LSTM aggregator and a transformer for spatial tile fusion, reporting leading results on the FIgLib dataset. Yar et al. [7] introduce a modified YOLOv5 for smart cities. Segmentation-based approaches extend detection to pixel-level fire localisation [30], though at higher computational cost. A shared limitation of this body of work is that performance figures are tied to dataset-specific benchmarks, making direct cross-paper comparisons unreliable [31,32,33]. NexFire Pro does not propose a new detector architecture; it asks how much of the residual operational error can be removed by lightweight, interpretable pipeline stages around a fixed backbone.

### 2.2. Night-Vision Enhancement Techniques

Detecting fire under low-light or near-infrared conditions is a known weakness of models trained on daytime visible-spectrum data [34,35]. When cameras switch to IR mode, images collapse toward near-grayscale, removing spectral cues on which colour-based detectors rely. CLAHE improves local contrast without disproportionate noise amplification [36], and pairing it with gamma correction for global brightness adjustment is a widely adopted strategy [37]. Learning-based enhancement networks achieve stronger benchmark results [38,39,40] but introduce a second GPU-bound inference stage that is difficult to justify in a system already handling sixteen concurrent streams.

### 2.3. Background Subtraction and Motion Validation

The MOG2 Gaussian mixture model [41] is widely deployed for foreground detection because it adapts incrementally to gradual scene changes and handles multimodal backgrounds effectively. Garcia Garcia et al. [42] document its failure modes under severe illumination shifts but note its continued competitiveness in relatively static industrial scenes. Incorporating motion cues as a secondary filter was explored in early fire-detection work [10,11,12] and revisited in neural detection pipelines: the FLAME framework [43] explicitly combines deep detection with motion analysis. The present system adopts a lighter approach using per-detection motion density computed from MOG2 foreground masks.

### 2.4. Region-of-Interest Management

ROI-based filtering is standard in surveillance applications where parts of the scene contain expected heat sources [13,16]. Using normalised polygon coordinates rather than absolute pixel offsets ensures configuration portability across cameras of different resolutions. This aspect has been explored in fixed-camera fire-surveillance systems [44] and video surveillance systems more broadly [45], but its integration into the full detection pipeline of a multi-camera desktop system has received limited attention.

### 2.5. Temporal Confirmation and Alarm Hysteresis

Alarm stability in video-based detection systems is commonly improved by requiring consistent detection across a temporal window before triggering [14,15]. A complementary grace period prevents rapid toggling when the detector oscillates between states on consecutive frames [15]. NexFire Pro realises this behaviour through per-class onset and recency timers that expose two intuitive parameters for per-camera tuning.

### 2.6. Multi-Camera Surveillance Systems

Scaling video analytics to many simultaneous streams is as much a resource management problem as an algorithmic one. BiSwift [46] and CANS [47] explore bandwidth and quality-of-service-aware scheduling. In practice, one processing thread per stream combined with adaptive frame skipping has proven effective on commodity hardware [48]. NexFire Pro follows this pattern, using a dedicated acquisition thread per camera with a configurable skip factor set automatically by source type.

## 3. System Architecture

### 3.1. Overview

**Figure 1:**
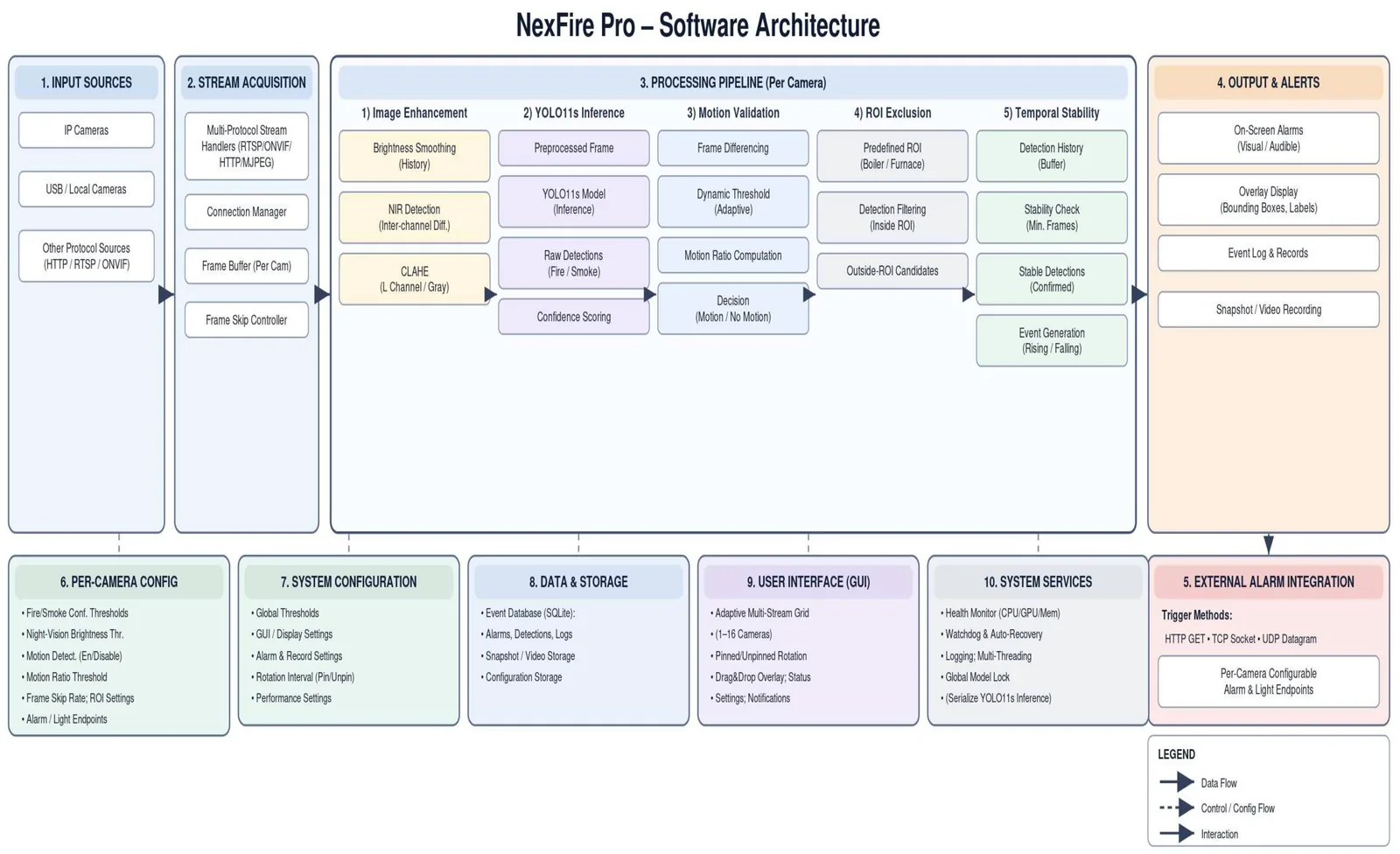
Overall software architecture of NexFire Pro. Heterogeneous input sources—IP cameras, USB/local cameras, and other RTSP/ONVIF/HTTP/MJPEG streams (1)—are ingested by the stream-acquisition layer (2) and processed per camera through the five-stage detection pipeline (3): image enhancement (brightness smoothing, near-infrared detection, and CLAHE), YOLO11s inference, motion validation, ROI exclusion, and temporal-stability confirmation. Confirmed detections drive on-screen alarms, overlay display, event logging, and snapshot/video recording (4), with optional external alarm integration over HTTP, TCP, or UDP (5). Supporting components include per-camera and system configuration (6, 7), SQLite-based data and storage (8), the adaptive multi-stream graphical interface (9), and background system services such as health monitoring, watchdog and auto-recovery, logging, and the global model lock (10). Solid arrows denote data flow; dashed lines denote control and configuration flow.

NexFire Pro is implemented in Python 3.10 using PyQt5 for the graphical interface, OpenCV 4.8 for video capture and classical image processing, and the Ultralytics YOLO11 framework (Ultralytics 8.x) for neural inference [49]. The system is organised into three loosely coupled subsystems—stream acquisition, the detection pipeline, and the user interface—which interact through well-defined interfaces and can be tested or modified independently (Figure 1).

### 3.2. Multi-Threaded Stream Acquisition

Camera streams are abstracted behind a unified acquisition interface that hides protocol-specific initialisation. For network sources RTSP, ONVIF, MJPEG, and HLS, the underlying FFmpeg backend is configured for connection stability using TCP transport and extended timeout values. Local USB cameras are initialised through platform-appropriate backends with MJPEG encoding enforced to eliminate intermittent blank frame artefacts observed on Windows systems. Each camera runs in a dedicated processing thread with automatic reconnection and exponential back off.

### 3.3. Detection Pipeline

Each incoming frame traverses five sequential stages—illumination enhancement, neural detection, motion validation, ROI exclusion, and temporal hysteresis—before an alarm decision is reached. The MOG2 foreground mask is computed from the frame and is used only to validate detector outputs, so motion validation necessarily follows neural detection; the complete per-frame procedure is given in Algorithm 1. The detection confidence score is one input to the alarm logic, not its sole determinant; each upstream and downstream stage acts as an independent filter. This cascaded design keeps individual stages simple and, as the ablation study in Section 5.2 demonstrates, distributes error suppression across qualitatively distinct failure modes.
**Algorithm 1.** Per-frame processing for camera c.Input: Frame f from camera c; per-camera configuration (thresholds τ, ROI polygons, T_onset, T_grace)Output: Alarm state for camera c (raised, cleared, or unchanged); overlay and event-log entry1:  f_e ← illumination_enhancement(f)       // night/IR classification, CLAHE, gamma2:  M ← MOG2_foreground_mask(f) 
         // frame-level background subtraction3:  D ← YOLO11s_detect(f_e) 
           // candidate boxes with class and score4:  for each box b in D do5:    if motion_density(b, M) < ρ_eff(b) then discard b    // motion validation6:  end for7:  for each surviving box b do8:    if centre(b) ∈ ROI exclusion zone then discard b    // ROI exclusion9:  end for10:  
update temporal-hysteresis state; raise or clear alarm using onset and grace-period rules11:  
emit overlay, event log, and external alarm outputs12:  
return alarm state for camera c

All key hyperparameters were selected through a two-stage process. In stage one, candidate ranges were drawn from the literature: CLAHE clip limit 2.0–4.0 following Zuiderveld [36], MOG2 history 300–700 frames following Zivkovic [41], and motion density threshold range 0.05–0.30 following practice in motion-filtered detection work [50]. In stage two, values were chosen on the training/validation data only and then locked before any use of the leakage-free held-out test set. The final selected values are: CLAHE clip limit 3.0, MOG2 history 500 frames, motion density threshold 0.15, and IR inter-channel-deviation threshold 5.0. Detector confidence operating points are reported explicitly in Section 5.2 as a sensitivity/operating-point sweep rather than as a single tuned value, so that pipeline gains are not confounded with threshold tuning.

## 4. Implementation Details

### 4.1. Night-Vision Enhancement

The illumination module classifies each frame into one of three modes using two criteria applied to a 40 × 40 downsampled representation. Let $\bar{\mu }$ denote the smoothed mean brightness over the *N* = 10 most recent frames and let

Δ = mean(|B − G|) + mean(|G − R|)(1)

inter-channel colour deviation. A frame is classified as IR mode when Δ < δ_IR (δ_IR = 5.0); as low-light colour mode when Δ ≥ δ_IR and $\bar{\mu }$ < θ_low (θ_low = 80 on a [0, 255] scale); and as standard colour mode otherwise. Threshold values were selected by the two-stage procedure described in Section 3.3. Because this mode assignment is derived directly from the pixel data rather than from any per-camera setting, infrared and conventional colour cameras can be mixed freely within a single deployment: each stream is assigned its enhancement mode automatically at runtime, with no manual camera-type configuration or day/night scheduling required. This is operationally relevant because real installations routinely combine fixed visible-spectrum cameras with IR-capable units that switch to near-grayscale output after dark.

For near-grayscale (IR-mode) inputs, Contrast Limited Adaptive Histogram Equalisation (CLAHE) with a clip limit of c_l = 3.0 and an 8 × 8 tile grid is applied to the single-channel intensity image. The clip limit of 3.0 is the midpoint of the 2.0–4.0 range recommended by Zuiderveld [36] for medical imaging applications with comparable signal-to-noise characteristics; values below 2.0 produce visible contrast plateaus on near-IR fire targets, and values above 4.0 amplify sensor noise to the point that motion-validation false-positive rates increased measurably during the threshold-selection grid search reported in Section 3.3. The 8 × 8 tile grid follows the OpenCV reference implementation default and matches the tile granularity recommended for surveillance-resolution input in [37]. For low-light colour inputs (Δ ≥ δ_IR and $\bar{\mu }$ < θ_low), enhancement is performed in the CIE L*a*b* colour space: only the L* (luminance) channel is processed with CLAHE, leaving the a* and b* chromaticity channels unchanged. Preserving colour fidelity matters because the YOLO11s detector relies partly on the characteristic orange-red spectral signature of flames. Adaptive gamma correction is then applied to the enhanced channel as I_out(x,y) = 255 × (I_in(x,y)/255)^{1/γ}, where I_in(x,y) ∈ [0, 255] is the pixel intensity before correction, I_out(x,y) is the corrected intensity, and γ is selected from the set {1.5, 1.8, 2.0} as a piecewise function of $\bar{\mu }$: γ = 2.0 if $\bar{\mu }$ < 40, γ = 1.8 if 40 ≤ $\bar{\mu }$ < 60, and γ = 1.5 if $\bar{\mu }$ ≥ 60 (thresholds on the [0, 255] scale). These breakpoints were fixed during the hyperparameter grid search described in Section 3.3.

### 4.2. Neural Detection

An initial gate threshold τ_0_ = 0.20 passes a broad candidate set to the downstream filters. Per-class base thresholds τ_fire = 0.40 and τ_smoke = 0.45 are then applied; both are user-configurable. Two runtime adjustments lower the effective threshold for high-difficulty detections: τ_eff ← τ_base − 0.12 under IR mode (compensating for reduced spectral contrast) and a further − 0.08 for bounding boxes in the upper region of the frame (accommodating low-confidence early-stage smoke). Both may apply simultaneously. Neural inference is serialised across acquisition threads using a global lock to prevent CUDA race conditions. The compact YOLO11s backbone (9.41 M parameters, 21.3 GFLOPs) also permits CPU-only deployment at reduced throughput when GPU resources are unavailable [51].

### 4.3. Region-of-Interest Filtering

User-defined exclusion zones are stored as ordered lists of normalised polygon vertices with coordinates in the range [0, 1] relative to frame dimensions. At inference time, these coordinates are scaled to the 640 × 640 model input space. For each surviving bounding box, the centre point is tested against all exclusion polygons using a point-in-polygon algorithm; boxes whose centres fall within an exclusion zone are removed before the temporal consistency check. Normalised coordinates make stored zone definitions portable across cameras of different resolutions and decouple the polygon drawing interface from the detection backend.

### 4.4. Temporal Consistency and Alarm Hysteresis

Each camera maintains independent state objects for the fire and smoke classes. Each state object records the timestamp of the first detection in the current run and the timestamp of the most recent detection. An alarm is raised only when the elapsed time since the first detection exceeds an onset threshold (T_onset = 2.0 s by default; configurable range 0.5–10.0 s). A grace period of T_grace = 0.5 s (configurable range 0.1–5.0 s) prevents rapid alarm toggling. These two parameters are exposed through the per-camera configuration dialog, allowing operators to tune the sensitivity–stability trade-off for specific environments. One additional runtime adjustment is applied: when the current frame is classified as IR mode (is_night = True), the effective onset threshold is reduced to T_onset,eff = T_onset × 0.6. For the default T_onset = 2.0 s, this yields T_onset,eff = 1.2 s under night conditions. The rationale is that low-light detection sequences are typically more fragmented due to lower model confidence and sparser motion masks, so requiring the full 2.0 s before confirmation would systematically delay night-mode alarms. The grace period T_grace is not adjusted for night mode.

Formally, let t_first and t_last denote the timestamps of the first and most recent detections in the current run on a given camera and class. An alarm is raised when

t_last − t_first ≥ T_onset(2)

The alarm is cancelled when the inter-detection gap exceeds T_grace. Isolated transient detections shorter than T_onset are suppressed, and a confirmed alarm does not toggle off on a single missed frame.

### 4.5. Per-Camera Configuration and External Alerting

A per-camera settings dialog exposes independent control over fire and smoke confidence thresholds, night-vision activation luminance, motion detection enablement and density threshold, and the frame skip factor. Local USB cameras operate at full frame rate (skip factor = 1), while network streams default to a skip factor of 5, yielding an effective analysis rate of approximately 12–15 FPS against a 60 FPS input stream. Outbound alerting supports HTTP GET, TCP socket, and UDP datagram communication, enabling integration with heterogeneous industrial alarm infrastructure.

### 4.6. Adaptive Display Layout

An adaptive grid layout module determines the optimal camera arrangement for between one and sixteen simultaneous feeds using ceiling-based square root decomposition, ensuring balanced spatial distribution. A subset of cameras can be designated as fixed position, remaining in assigned grid slots across layout rotation cycles, while others rotate through remaining positions at a configurable interval (default: 20 s). A draggable summary overlay aggregates active detection events from all cameras, providing operators with a consolidated situational view without requiring them to scan all sixteen panels.

## 5. Experimental Evaluation

### 5.1. Dataset and Experimental Setup

All experiments were conducted on a Windows 10 workstation with an Intel Core i7-12700K processor, 32 GB of DDR4-3200 RAM, and an NVIDIA GeForce RTX 3060 GPU (12 GB VRAM). The deployment uses a small number of physical cameras—a Hikvision ColorVu infrared dome camera and a laptop USB webcam (Hikvision Digital Technology Co., Ltd., Hangzhou, China)—together with public Internet IP-camera streams (RTSP/HTTP/MJPEG) and pre-recorded fire/smoke video files connected to the software’s stream channels. Because the program is fully dynamic, the same detection pipeline is applied identically to every channel regardless of source; multi-source operation is therefore a replication of the same processing across concurrent streams rather than a fixed physical camera array. The IR/night regime is entered automatically by the illumination module using a fixed inter-channel-deviation threshold (Δ < 5.0), set before evaluation and not tuned to outcomes.

#### Training Dataset

The detector was trained on labelled fire/smoke imagery under a single consistent two-class annotation. A source-disjoint, leakage-free held-out test set was reserved for evaluation: duplicate and near-duplicate frames shared with the training data were removed so that no test image (or a near-duplicate of it) appears in training. Public still-image benchmarks were used for training only; the held-out test set is independent of them.

The held-out test set is kept independent of the training data to avoid the optimistic bias that frame-level splitting introduces when temporally adjacent, near-identical frames are placed on both sides of the split [17]. Concretely, the test set is disjointed by the source from the training pool, leaving 249 leakage-free test images. All reported detection figures use only this locked test set. We do not claim a conventional physical-camera k-fold: the deployment uses only a small number of physical cameras plus Internet streams and pre-recorded clips, so balanced multi-camera cross-validation is not meaningful here; instead, we rely on strict source disjointness and near-duplicate removal. Where a public source exposes sequence or fire-event identifiers (for example, the per-event sequences in FIgLib), frames from the same sequence are kept within a single split to prevent near-duplicate leakage.

The detection backbone is YOLO11s (Ultralytics) in its default configuration: a CSPDarknet-derived backbone with C3k2 blocks and SiLU activations, a path-aggregation feature-pyramid neck, and a decoupled anchor-free detection head. The model has approximately 9.41 million parameters and an inference cost of 21.3 GFLOPs. Training started from COCO-pretrained weights using the Ultralytics implementation: an SGD-family optimiser, batch size 16, input image size 896, up to 200 epochs with early stopping (patience 30), and augmentation comprising mosaic, horizontal flip, light HSV jitter, and scaling. On the RTX 3060, the model runs at approximately 8.3 ms per frame (~120 FPS). The trained weights, training configuration, evaluation scripts, and the data-split manifest are released alongside the source code (see Data Availability).

### 5.2. Ablation Study

To isolate each stage’s contribution, we used a leave-one-out design: starting from the complete pipeline, each stage (motion validation, night-vision preprocessing, temporal hysteresis) was removed individually; ROI exclusion is scene-dependent and was not separately quantified on the evaluated clips, and we additionally evaluated a detection-only baseline with a single fixed confidence threshold applied to all classes (addressing the threshold-confound concern). All configurations share the same trained YOLO11s weights and were run on the same non-fire video, where every raised alarm is, by definition, a false alarm. Frame-level detections are consolidated into alarm events by the temporal-hysteresis stage: an alarm event begins at onset confirmation and absorbs subsequent detections of the same class on the same camera separated by less than the grace period, so a single sustained episode produces exactly one alarm event, and repeated frame-level firings are not double-counted. We report the absolute number of false-alarm events per configuration; where the recording duration allows, we also express this as false alarms per stream-hour.

The ordering of configurations in Table 1 follows the position of each stage in the runtime pipeline (preprocessing → neural detection → motion validation → ROI exclusion → temporal hysteresis). Night-vision preprocessing primarily improves recall under low-light conditions and is characterised separately in Section 5.3. Because each configuration removes exactly one stage from the otherwise complete pipeline while all other stages remain active, the reported increase in false alarms isolates the independent contribution of the removed stage. The stages were designed around largely disjoint failure modes (static bright objects, fixed structural hot zones, near-infrared degradation, and transient single-frame flicker), which is consistent with the pattern of contributions observed in Table 1.

It should be noted that this leave-one-out ablation was conducted on non-fire footage only, where every alarm is by definition a false alarm. Fire event recall for each ablated configuration was not separately measured; fire recall is reported at the operating-point level in Table 2.

On external video (Bilkent VisiFire), the end-to-end system reaches an event-level recall of 44.4% at the conservative operating point and 61.1% at the sensitive operating point, at the cost of correspondingly more false alarms on non-fire footage (Table 2).

Table 3 reports overall and per-class detection metrics on the leakage-free test set. Per-class detection results are: fire—precision 0.727, recall 0.536, mAP@0.5 = 0.551; smoke—precision 0.801, recall 0.746, mAP@0.5 = 0.758. Smoke is detected more reliably than fire on this set; overall mAP@0.5 is 0.654 (mAP@0.5:0.95 = 0.383, precision 0.764, recall 0.641, F1 0.697).

These qualitative cases map onto the leave-one-out ablation (Table 1). Case (a) corresponds to motion validation—the stage responsible for the largest reduction in false alarms, since a static fire-coloured region with no foreground motion is rejected. Case (b) corresponds to ROI exclusion, which removes detections falling inside user-defined ignore zones. Cases (c)–(d) correspond to the detection and night-vision stages. Each visual failure mode therefore aligns with a specific, interpretable stage of the pipeline rather than an incidental test-set property.

### 5.3. Night-Vision Performance

A dedicated labelled near-infrared benchmark is identified as future work; the night-vision stage (automatic IR-mode detection and CLAHE-based enhancement) is described here as an implementation feature, to be evaluated per sensor and scene. In the leave-one-out ablation on the available (daytime) non-fire footage, disabling night-vision preprocessing did not change the false-alarm count.

To quantify labelling consistency, an independent second annotator relabelled an 80-image subset of the evaluation frames, blind to the original labels and following the same predefined guidelines. On the matched detections, the two annotators showed substantial class-level agreement (Cohen’s κ = 0.75; 91.5% raw agreement on the fire-versus-smoke decision), with a mean localisation overlap of IoU = 0.65. Box-level detection agreement was lower (F1 = 0.51), indicating that the principal differences lay in which faint or boundary regions each annotator chose to mark rather than in the class assigned to the regions they agreed on. All bounding boxes in the primary dataset were drawn at visible fire or smoke boundaries by a domain expert with five or more years of industrial surveillance experience, following the same guidelines.

### 5.4. Parameter Sensitivity Analysis

Rather than a single tuned detector confidence, operating points are reported explicitly as a sensitivity/operating-point sweep (Section 5.2, Table 2), so that the pipeline’s contribution is not confounded with threshold tuning; a dedicated per-threshold sensitivity table was not conducted for this revision.

### 5.5. Failure Cases and System Limitations

**Figure 2:**
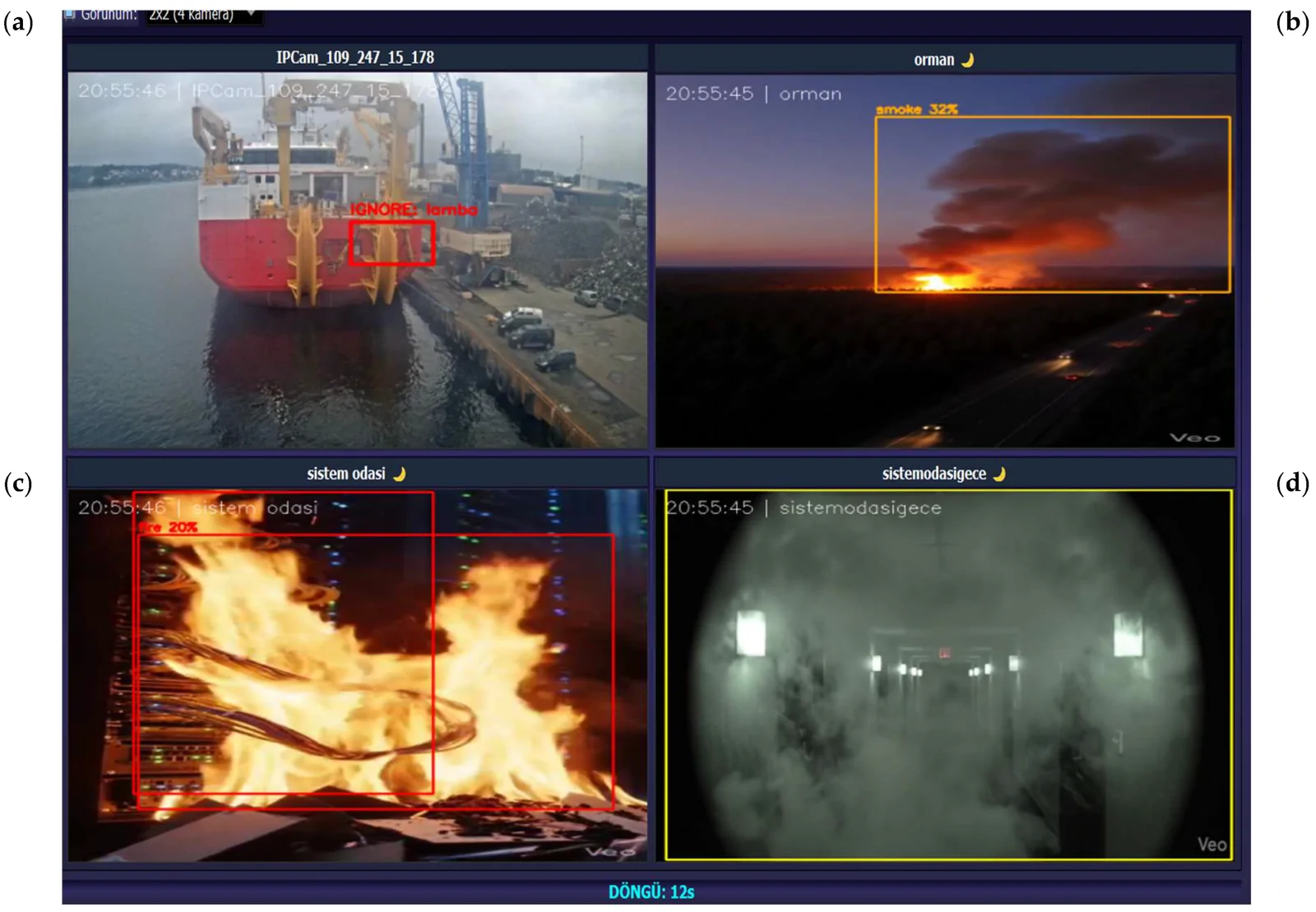
Qualitative evaluation of the NexFire Pro pipeline under four representative failure scenarios addressed by the system. (**a**) Vehicle headlight false positive: baseline triggers alarm; motion validation correctly suppresses it (no foreground motion in bounding box). (**b**) Sun-reflection false positive: baseline triggers alarm; ROI exclusion removes the persistent activation from the known hot zone. (**c**) Small early-stage flame: the baseline misses detection at low confidence; the night-vision preprocessing stage recovers it under mixed illumination. (**d**) Near-IR night frame: raw IR input produces missed smoke detection; CLAHE enhancement restores local contrast and enables correct identification. Bounding boxes: yellow = correct detection, red = false positive, dashed red = missed detection recovered by pipeline. Panel-to-stage correspondence and the associated false-alarm reductions are quantified in Table 1 (Section 5.2).

Figure 2 illustrates qualitatively four representative failure scenarios addressed by the pipeline; the failure modes discussed below extend beyond those four cases to the broader range of conditions encountered during real-world deployment.

### 5.6. Long-Duration Operational Stability

Long-duration operational stability over a continuous 8.38 h run (host-level CPU/RAM/GPU telemetry) is characterised in the Supplementary Material (Tables S1–S5 and Figure S1).

## 6. Discussion

In the leave-one-out ablation, motion validation accounts for the largest share of the false-alarm reduction: removing it lets the majority of the suppressed false alarms through. This aligns with the established finding that static or slowly changing bright objects—indicator lights, furnace glows, sun reflections—are the primary source of false positives in fire-like scenes [15,16]; requiring foreground presence eliminates them without affecting genuinely dynamic events. Temporal hysteresis removes short, sub-onset-duration flickering, consistent with temporal-consistency mechanisms described in [15]. ROI exclusion is relevant only for cameras with structurally hot zones and was not separately quantified on the evaluated clips. Reducing spurious alarms is operationally meaningful: alarm fatigue—the tendency of operators to discount repeated false alerts—is a documented risk in continuous monitoring environments [3,52], so improving precision increases the probability that a genuine event receives prompt attention.

IR/night-mode performance is rarely documented in the fire-detection literature, despite IR operation being the default for most industrial cameras overnight. NexFire Pro includes an automatic IR-mode detector and CLAHE-based enhancement for this regime; a quantitative near-infrared evaluation on a labelled IR set is identified as important future work and should be validated per sensor and scene before any night-mode gain is claimed.

A broader implication of these results is that, in safety-critical vision systems, operational reliability can depend as much on how the pipeline is designed and evaluated as on the detector itself. Three elements of our approach work toward this together: tying each stage to a specific failure mode, evaluating under a leakage-free, source-disjoint split, and quantifying each stage’s contribution through ablation. We see this combination as a reusable way to study reliability in such systems and expect it to transfer to neighbouring problems such as industrial monitoring, anomaly detection, and intelligent surveillance more broadly. This perspective complements architecture-centric research by emphasising reproducible evaluation methodology alongside detector innovation.

Furthermore, although the related work situates NexFire Pro against recent fire- and smoke-detection methods—including the transformer-based SmokeyNet, recent YOLO-family detectors (EFSD-YOLO, DSS-YOLO, and YOLOv7/v8/v9 variants), an EfficientDet-based wildfire-smoke detector, and SE-ResNet and CNN-based smoke detectors—along the dimensions of input modality, temporal reasoning, false-alarm handling, and multi-stream operation, a direct quantitative comparison on a single shared benchmark was not performed in the present study. Because these methods were trained and evaluated on different datasets, a statistically valid head-to-head comparison would require re-implementing or retraining each method under identical data, metric, and hardware conditions; we therefore regard this controlled re-evaluation as an important direction for future work. To facilitate it, we release our locked leakage-free, source-disjoint held-out partition together with the evaluation scripts, so that subsequent studies can benchmark other detectors under exactly the conditions reported here. Finally, inter-annotator agreement was assessed on an 80-image subset through an independent second annotation, yielding substantial class-level agreement (Cohen’s κ = 0.75; 91.5% agreement on the fire-versus-smoke decision); extending double annotation to the full evaluation set remains future work.

## 7. Conclusions

This paper presented NexFire Pro, a modular framework for real-time fire and smoke detection across multiple simultaneous camera streams. The system integrates a fine-tuned YOLO11s detector—trained under a single consistent two-class annotation with a source-disjoint, leakage-free held-out test set—and a five-stage post-detection pipeline consisting of illumination-aware night-vision enhancement, neural detection, MOG2-based motion validation, normalised-coordinate ROI filtering, and temporal alarm hysteresis.

On a leakage-free held-out test set, the detector attains mAP@0.5 = 0.65 (F1 = 0.70; precision 0.76, recall 0.64). In leave-one-out ablation on non-fire video, motion validation is the dominant false-alarm suppressor, reducing a raw-detector baseline of eight false alarms to zero, with temporal hysteresis removing a residual alarm. On external video, the end-to-end system reaches an event-level recall of 44–61% depending on the operating point, with a median time to first alarm of one to three seconds, and it runs at approximately 120 FPS single stream (neural inference only) on a single RTX 3060.

The results suggest that reliability-oriented pipeline engineering can deliver substantial operational gains in multi-camera fire surveillance systems, gains that are complementary to rather than in competition with architectural improvements to the underlying detector.

Finally, beyond the detection figures reported here, the most durable contribution of this work is methodological. Pairing failure-mode-driven pipeline design with a leakage-free evaluation and per-stage measurement yields reproducible evidence of how individual, interpretable components shape real-world performance—a perspective that complements advances in detector architecture and may serve as a reproducible framework for future research on safety-critical video analytics.

## Figures and Tables

**Table 1:** Independent (leave-one-out) false-alarm ablation on non-fire video; every raised alarm is by definition a false alarm.

Configuration	False-Alarm Events	Leave-One-Out Contribution
Baseline (detection only, fixed τ = 0.35)	8	—
Full pipeline—motion validation	5	+5 (dominant)
Full pipeline—temporal hysteresis	1	+1
Full pipeline—night enhancement	0	0
Full pipeline (all stages)	0	—

**Table 2:** Operating-point sweep: event-level recall vs. false alarms on external video (Bilkent VisiFire).

Operating Point	Event Recall (18 Clips)	False Alarms (Non-Fire, 79.6 s)
Conservative (fire 0.40/smoke 0.45/2.0 s)	8/18 = 44.4%	0
Sensitive (fire 0.25/smoke 0.30/1.0 s)	11/18 = 61.1%	2

**Table 3:** Detection metrics on the leakage-free held-out test set (249 source-disjoint images, image size 896).

Class	Precision	Recall	F1	mAP@0.5	mAP@0.5:0.95
All	0.764	0.641	0.697	0.654	0.383
Fire	0.727	0.536	0.617	0.551	—
Smoke	0.801	0.746	0.773	0.758	—

## Data Availability

Upon publication, the following materials will be released in a public repository: the trained YOLO11s model weights, the training configuration, the leakage-free data-split manifest (with the near-duplicate-removal list defining the held-out test set), the detection-evaluation script (order-safe, class-name-aware) and the headless pipeline-evaluation harness used for the ablation and operating-point experiments, the list of connectable camera/stream sources, and the host-level telemetry logs from the long-duration stability run. Until then, these materials are available from the corresponding author upon reasonable request.

## Supplementary Materials

The following supporting information can be downloaded at: https://www.mdpi.com/article/10.3390/s26165038/s1, Table S1: CPU utilisation statistics over the 8.38-h stability session. All values in percent (%). Variance unit: %^2^. Table S2: RAM allocation statistics over the 8.38-h stability session. All values in MB. Linear regression slope: 94.19 MB/h (Pearson r = 0.868, *p* < 0.001). Variance unit: MB. Table S3: GPU compute utilisation statistics over the 8.38-h stability session. All values in percent (%). Variance unit: %. Table S4: GPU core temperature statistics over the 8.38-h stability session. All values in °C. Temperature–clock correlation r = −0.008 (no thermal throttling detected). Variance unit: °C. Table S5: Drift analysis: comparison of first 10% vs. last 10% session averages for each monitored resource. All four resources are classified as Stable, confirming long-duration operational equilibrium under the eight-stream 1080p workload. Figure S1: Long-duration operational stability over the 8.38-h session (22:36–06:59). (a) CPU utilisation (%) over 503 min; mean 21.94%, max 44.40%, std 2.43%. (b) RAM allocation (MB) over time; upward trend slope 94.19 MB/h (r = 0.868). (c) GPU compute load (%) over 503 min; mean 24.14%, max 47.20%. (d) GPU core temperature (°C); mean 60.30 °C, max 66.09 °C, std 1.04 °C. (e) VRAM consumption (MB); stable oscillation between 3800–4500 MB. (f) CPU clock frequency (MHz); constant at ~1400 MHz throughout—no throttling. (g) GPU core clock (MHz); sustained ~1800 MHz with no sustained downward drift. (h) RAM linear regression fit (slope 94.19 MB/h); 24-h projection ≈ 2260 MB, within system capacity. (i) GPU temperature vs. clock frequency (throttling check); correlation r = −0.008, confirming absence of thermal throttling. (j) First 10% vs. last 10% mean comparison for CPU, RAM, GPU, and temperature; all resources stable. (k) Long-duration parameter statistics: mean, median, min, max, standard deviation, and variance for CPU utilisation, RAM allocation, GPU utilisation, and GPU core temperature over the 8.38-h session. (l) Long-duration drift analysis: first 10% vs. last 10% session averages for each resource metric, with absolute and percentage change.

## Abbreviations

The following abbreviations are used in this manuscript:

AI	Artificial Intelligence
CLAHE	Contrast Limited Adaptive Histogram Equalization
F1	F1 Score
FIgLib	Fire Ignition Library
FPS	Frames Per Second
GPU	Graphics Processing Unit
HSV	Hue Saturation Value (colour space)
HTTP	Hypertext Transfer Protocol
IP	Internet Protocol
IR	Infrared
mAP	Mean Average Precision
MJPEG	Motion JPEG
MOG2	Mixture of Gaussians (background subtraction algorithm)
ONVIF	Open Network Video Interface Forum
ROI	Region of Interest
RTSP	Real-Time Streaming Protocol
TCP	Transmission Control Protocol
UDP	User Datagram Protocol
USB	Universal Serial Bus
YOLO	You Only Look Once

## References

[1] National Fire Protection Association (NFPA) (2023). NFPA Report: Industrial and Manufacturing Properties, 2017–2021.

[2] Çelik T., Demirel H. (2009). Fire detection in video sequences using a generic color model. Fire Saf. J..

[3] Festag S. (2016). False alarm ratio of fire detection and fire alarm systems in Germany—A meta analysis. Fire Saf. J..

[4] Bu F., Gharajeh M.S. (2019). Intelligent and vision-based fire detection systems: A survey. Image Vis. Comput..

[5] Redmon J., Divvala S., Girshick R., Farhadi A. (2016). You Only Look Once: Unified, real-time object detection. Proceedings of the IEEE/CVF CVPR.

[6] Liu Z., Ma X., Feng Y., Sun J. (2025). DSS YOLO: An improved lightweight real-time fire detection model based on YOLOv8. Sci. Rep..

[7] Yar H., Khan Z.A., Ullah F.U.M., Ullah W., Baik S.W. (2023). A modified YOLOv5 architecture for efficient fire detection in smart cities. Expert Syst. Appl..

[8] Chino D.Y.T., Avalhais L.P.S., Rodrigues J.F., Traina A.J.M. (2015). BoWFire: Detection of fire in still images by integrating pixel color and texture analysis. Proceedings of SIBGRAPI.

[9] Islam A., Habib M.I. (2023). Fire detection from image and video using an improved YOLOv5 model. arXiv.

[10] Vijayalakshmi S.R., Muruganand S. (2017). Smoke detection in video images using background subtraction method for early fire alarm system. Proceedings of the 2017 2nd International Conference on Communication and Electronics Systems (ICCES), Coimbatore, India, 19–20 October 2017.

[11] Filonenko A.A., Cullip D.A., Jo K.H. (2018). Fast smoke detection for video surveillance using CUDA. IEEE Trans. Ind. Inform..

[12] Hashemzadeh M., Farajzadeh N., Heydari M. (2022). Smoke detection in video using convolutional neural networks and efficient spatio-temporal features. Appl. Soft Comput..

[13] Bugarić M., Jakovčević T., Stipaničev D. (2014). Adaptive estimation of visual smoke detection parameters based on spatial data and fire risk index. Comput. Vis. Image Underst..

[14] Töreyin M., Dedeoğlu Y., Çetin A.E. (2005). Flame detection in video using hidden Markov models. Proceedings of IEEE ICIP.

[15] Gomes P., Santana P., Barata J. (2014). A vision based approach to fire detection. Int. J. Adv. Robot. Syst..

[16] Töreyin B.U., Dedeoğlu Y., Güdükbay U., Çetin A.E. (2006). Computer vision based method for real-time fire and flame detection. Pattern Recognit. Lett..

[17] Dewangan A., Pande Y., Braun H.-W., Vernon F., Perez I., Altintas I., Cottrell G.W., Nguyen M.H. (2022). FIgLib & SmokeyNet: Dataset and deep learning model for real-time wildland fire smoke detection. Remote Sens..

[18] Zhou Y., Song X., Luo X., Gong Y. (2025). EFSD-YOLO: Fire and smoke detection based on enhanced YOLOv11. Proceedings of the 2025 4th International Conference on Intelligent Mechanical and Human-Computer Interaction Technology (IHCIT), Dalian, China, 22–24 August 2025.

[19] Çelik T., Demirel H., Özkaramanlı H., Uyguroğlu M. (2006). Fire detection in video sequences using statistical color model. Proceedings of the 2006 IEEE International Conference on Acoustics, Speech and Signal Processing (ICASSP), Toulouse, France, 14–19 May 2006.

[20] Ateeq Z., Momani M. (2020). Wireless sensor networks using image processing for fire detection. Proceedings of the 2020 5th International Conference on Innovative Technologies in Intelligent Systems and Industrial Applications (CITISIA), Sydney, Australia, 25–27 November 2020.

[21] Gong F., Li C., Gong W., Li X., Yuan X., Ma Y., Song T. (2019). A real-time fire detection method from video with multifeature fusion. Comput. Intell. Neurosci..

[22] Ko B.C., Cheong K.H., Nam J.Y. (2010). Early fire detection algorithm based on irregular patterns of flames and hierarchical Bayesian networks. Fire Saf. J..

[23] Hentati M., Gargouri A., Hentati R. (2026). Real-time indoor early fire detection in industrial environments using optimized multi-sensor edge deep learning. Eng. Res. Express.

[24] Sohan M., Sai Ram T., Rami Reddy C.V., Jacob I.J., Piramuthu S., Falkowski-Gilski P. (2024). A review on YOLOv8 and its advancements. Data Intelligence and Cognitive Informatics.

[25] Fernandes A.M., Utkin A.B., Chaves P. (2023). Automatic early detection of wildfire smoke with visible-light cameras and EfficientDet. J. Fire Sci..

[26] Zell O., Pålsson J., Hernandez-Diaz K., Alonso-Fernandez F., Nilsson F. (2023). Image-based fire detection in industrial environments with YOLOv4. Proceedings of the 12th International Conference on Pattern Recognition Applications and Methods (ICPRAM 2023), Lisbon, Portugal, 22–24 February 2023.

[27] Zhang Z., Tan L., Tiong R.L.K. (2024). An improved fire and smoke detection method based on YOLOv8n for smart factories. Sensors.

[28] Zhang Y., Xiao X., Wang W., Wang C., Jin X., Wang Y. (2024). Improved lightweight flame smoke detection algorithm for YOLOv8n. Proceedings of the 2024 39th Youth Academic Annual Conference of Chinese Association of Automation (YAC), Dalian, China, 7–9 June 2024.

[29] Wang Z. (2024). Enhanced fire detection algorithm for chemical plants using modified YOLOv9 architecture. Proceedings of the 2024 9th International Conference on Electronic Technology and Information Science (ICETIS), Zhuhai, China, 17–19 May 2024.

[30] Carletti V., Greco A., Saggese A., Vento B. (2026). Real-Time Fire Detection From Video Surveillance Cameras: Where Are We Now?. IEEE Access.

[31] Gaur A., Singh A., Kumar A., Kumar A., Kapoor K. (2020). Video flame and smoke based fire detection algorithms: A literature review. Fire Technol..

[32] Gragnaniello D., Greco A., Sansone C., Vento B. (2024). Fire and smoke detection from videos: A literature review under a novel taxonomy. Expert Syst. Appl..

[33] Chaturvedi S., Khanna P., Ojha A. (2022). A survey on vision-based outdoor smoke detection techniques for environmental safety. ISPRS J. Photogramm. Remote Sens..

[34] Tatana M.M., Tsoeu M.S., Maswanganyi R.C. (2025). Low-light image and video enhancement for more robust computer vision tasks: A review. J. Imaging.

[35] Wu Y., Yin T., Zhao X. (2022). Using deep learning with thermal imaging for human detection in heavy smoke scenarios. Sensors.

[36] Zuiderveld K. (1994). Contrast Limited Adaptive Histogram Equalization. Graphics Gems IV.

[37] Gonzalez R.C., Woods R.E. (2018). Digital Image Processing.

[38] Weng S.-E., Miaou S.-G., Christanto R. (2025). A lightweight low-light image enhancement network via channel prior and gamma correction. Int. J. Pattern Recognit. Artif. Intell..

[39] Reis M.J.C.S. (2025). Low-light image enhancement using deep learning: A lightweight network with synthetic and benchmark dataset evaluation. Appl. Sci..

[40] Lore K.G., Akintayo A., Sarkar S. (2017). LLNet: A deep autoencoder approach to natural low-light image enhancement. Pattern Recognit..

[41] Zivkovic Z. (2004). Improved adaptive Gaussian mixture model for background subtraction. Proceedings of ICPR.

[42] Garcia Garcia B., Bouwmans T., Silva A.J.R. (2020). Background subtraction in real applications: Challenges, current models and future directions. Comput. Sci. Rev..

[43] Gragnaniello D., Greco A., Sansone C., Vento B. (2025). FLAME: Fire detection in videos combining a deep neural network with a model-based motion analysis. Neural Comput. Appl..

[44] Liu C.B., Ahuja N. (2004). Vision based fire detection. Proceedings of the IEEE ICPR.

[45] Quast K., Kaup A. (2008). Spatial scalable region of interest transcoding of JPEG2000 for video surveillance. Proceedings of the 2008 IEEE Fifth International Conference on Advanced Video and Signal Based Surveillance (AVSS), Santa Fe, NM, USA, 1–3 September 2008.

[46] Sun L., Wang W., Yuan T., Mi L., Dai H., Liu Y., Fu X. (2024). BiSwift: Bandwidth orchestrator for multi-stream video analytics on edge. Proceedings of IEEE INFOCOM 2024—IEEE Conference on Computer Communications, Vancouver, BC, Canada, 20–23 May 2024.

[47] Tu J., Xu Q., Chen C. (2021). CANS: Communication limited camera network self-configuration for intelligent industrial surveillance. Proceedings of the 2021 47th Annual Conference of the IEEE Industrial Electronics Society (IECON), Toronto, ON, Canada, 13–16 October 2021.

[48] Mukhiddinov M., Abdusalomov A.B., Cho J. (2022). A wildfire smoke detection system using unmanned aerial vehicle images based on the optimized YOLOv5. Sensors.

[49] Jocher G., Chaurasia A., Stoken A., Borovec J., NanoCode012, Kwon Y., Michael K., Xie T., Fang J., Imyhxy (2022). ultralytics/yolov5: v7.0—YOLOv5 SOTA Realtime Instance Segmentation.

[50] Xie Y., Li Z., Wang Q. (2019). An intelligent fire detection approach through cameras based on computer vision methods. Process Saf. Environ. Prot..

[51] de Venâncio P.V.A.B., Lisboa A.C., Barbosa A.V. (2022). An automatic fire detection system based on deep convolutional neural networks for low-power, resource-constrained devices. Neural Comput. Appl..

[52] Cvach M. (2012). Monitor alarm fatigue: An integrative review. Biomed. Instrum. Technol..

