# Supplementary material for: A Multi-Stage Computer Vision Framework for Real-Time Fire Detection in Low-Light Multi-Camera Environments
Source: Sensors (Basel). 2026 Aug 8;26(16):5038. doi: 10.3390/s26165038 (PMC13517262; doi:10.3390/s26165038)
Supplement: Supplementary file 1 [file sensors-26-05038-s001.zip › sensors-4415958-supplementary.pdf]

Supplementary Materials

A Multi-Stage Computer Vision Framework for Real-Time Fire Detection in Low-Light Multi-Camera Environments

Ruhi Taş

Department of Computer Engineering, OSTIM Technical University, Ankara 06374, Turkey; ruhi.tas@ostimteknik.edu.tr

This document contains the supplementary tables and figure referenced in the main manuscript. Tables S1–S5 and Figure S1 report detailed per-metric statistics and drift analysis from the 8.38-hour long-duration stability experiment described in the main text.

Table S1. CPU utilisation statistics over the 8.38-hour stability session. All values in percent (%). Variance unit: %².

Metric	Mean	Median	Min	Max	Std Dev	Variance
CPU Utilisation (%)	21.938	21.600	0.000	44.400	2.433	5.921

Table S2. RAM allocation statistics over the 8.38-hour stability session. All values in MB. Linear regression slope: 94.19 MB/h (Pearson $r = 0.868$, $p < 0.001$). Variance unit: MB².

Metric	Mean	Median	Min	Max	Std Dev	Variance
RAM Used (MB)	15,722.671	15,763.781	15,110.266	16,428.199	262.565	68,940.297

Table S3. GPU compute utilisation statistics over the 8.38-hour stability session. All values in percent (%). Variance unit: %².

Metric	Mean	Median	Min	Max	Std Dev	Variance
GPU Utilisation (%)	24.138	24.037	0.000	47.196	3.907	15.267

Table S4. GPU core temperature statistics over the 8.38-hour stability session. All values in °C. Temperature–clock correlation $r = -0.008$ (no thermal throttling detected). Variance unit: °C².

Metric	Mean	Median	Min	Max	Std Dev	Variance
GPU Temperature (°C)	60.302	60.278	55.830	66.091	1.039	1.078

Table S5. Drift analysis: comparison of first 10% vs. last 10% session averages for each monitored resource. All four resources are classified as Stable, confirming long-duration operational equilibrium under the eight-stream 1080p workload.

**Resource Metric**	**First 10% Avg**	**Last 10% Avg**	**Change**	**Status**
CPU Utilisation (%)	23.33	22.57	−3.23%	Stable
RAM Used (MB)	15,327.88	15,899.49	+3.73%	Stable
GPU Utilisation (%)	25.36	24.93	−1.66%	Stable
GPU Temperature (°C)	60.56	60.48	−0.08 °C	Stable

Figure S1

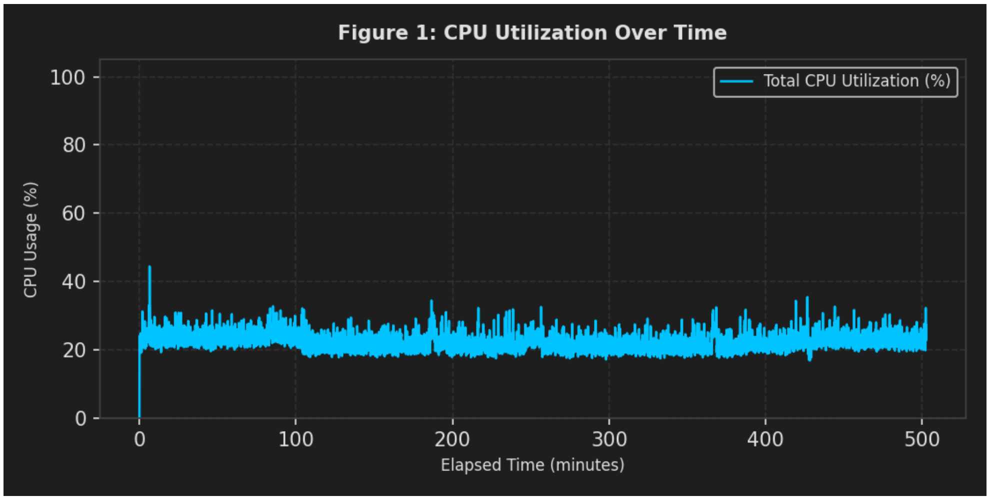

Figure 1: CPU Utilization Over Time
Total CPU Utilization (%)
CPU Usage (%)
100
80
60
40
20
0
0
100
200
300
400
500
Elapsed Time (minutes)

(a)

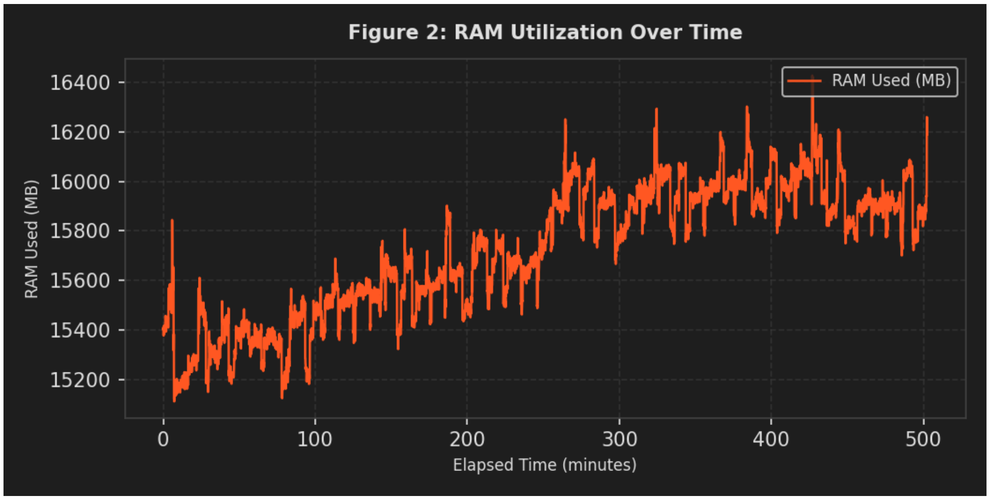

Figure 2: RAM Utilization Over Time
RAM Used (MB)
RAM Used (MB)
16400
16200
16000
15800
15600
15400
15200
0
100
200
300
400
500
Elapsed Time (minutes)

(b)

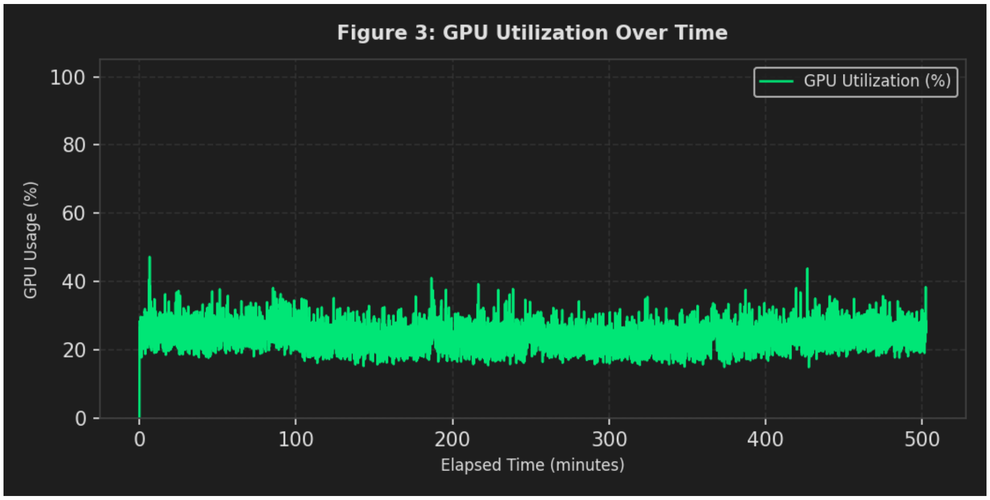
Figure 3: GPU Utilization Over Time
GPU Utilization (%)
GPU Usage (%)
100
80
60
40
20
0
0
100
200
300
400
500
Elapsed Time (minutes)

(c)

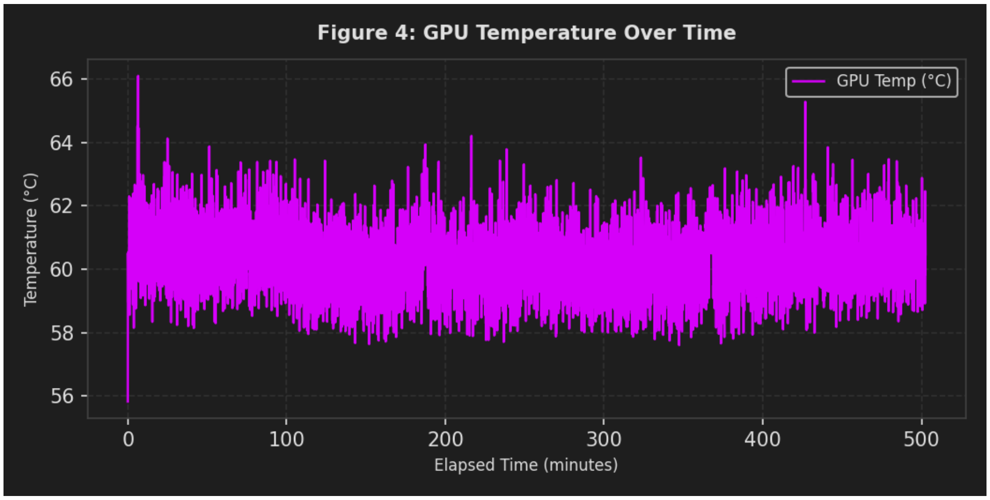
Figure 4: GPU Temperature Over Time
GPU Temp (°C)
Temperature (°C)
66
64
62
60
58
56
0
100
200
300
400
500
Elapsed Time (minutes)

(d)

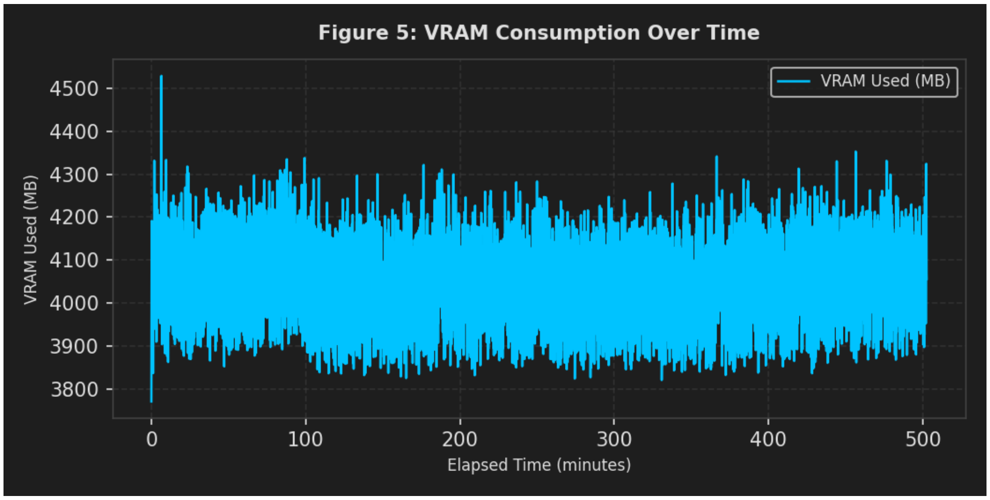
Figure 5: VRAM Consumption Over Time
VRAM Used (MB)
VRAM Used (MB)
4500
4400
4300
4200
4100
4000
3900
3800
0
100
200
300
400
500
Elapsed Time (minutes)

(e)

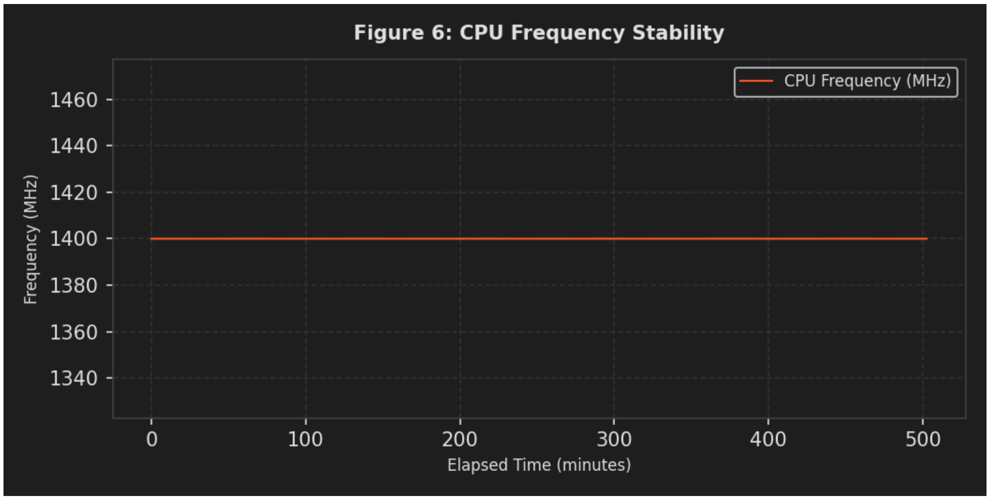
Figure 6: CPU Frequency Stability
CPU Frequency (MHz)
Frequency (MHz)
1460
1440
1420
1400
1380
1360
1340
0
100
200
300
400
500
Elapsed Time (minutes)

(f)

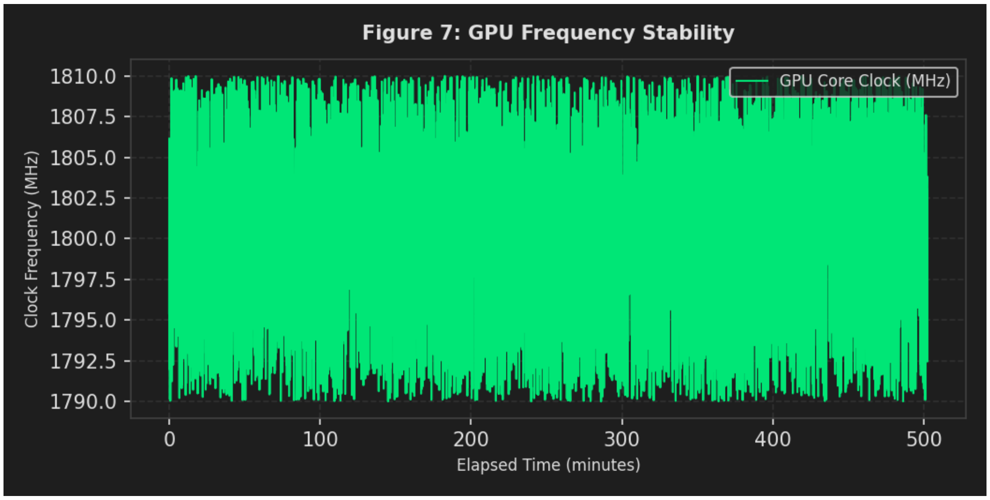
Figure 7: GPU Frequency Stability
GPU Core Clock (MHz)
Clock Frequency (MHz)
1810.0
1807.5
1805.0
1802.5
1800.0
1797.5
1795.0
1792.5
1790.0
0
100
200
300
400
500
Elapsed Time (minutes)

(g)

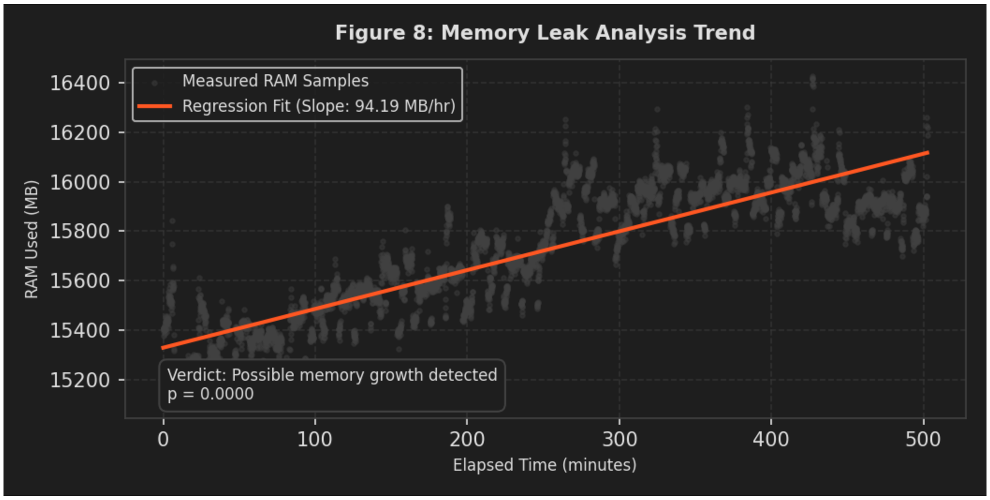
Figure 8: Memory Leak Analysis Trend
Measured RAM Samples
Regression Fit (Slope: 94.19 MB/hr)
Verdict: Possible memory growth detected
p = 0.0000
RAM Used (MB)
16400
16200
16000
15800
15600
15400
15200
0
100
200
300
400
500
Elapsed Time (minutes)

(h)

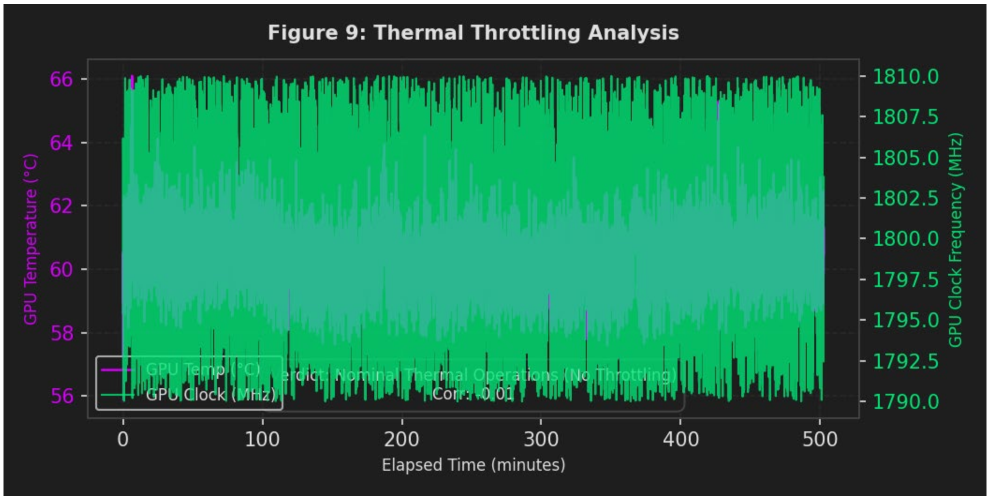
Figure 9: Thermal Throttling Analysis
GPU Temperature (°C)
66
64
62
60
58
56
GPU Clock Frequency (MHz)
1810.0
1807.5
1805.0
1802.5
1800.0
1797.5
1795.0
1792.5
1790.0
GPU Clock (MHz)
Nominal Thermal Operations (No Throttling)
0
100
200
300
400
500
Elapsed Time (minutes)

(i)

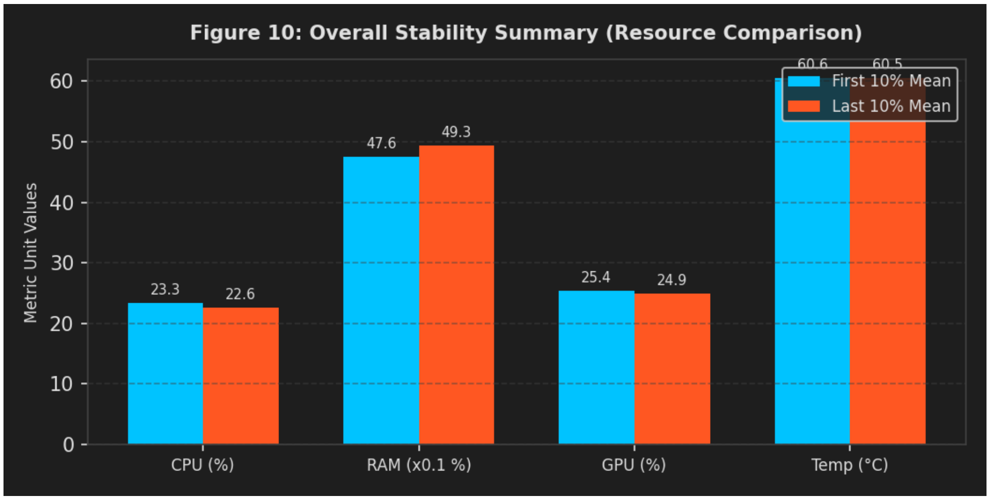
Figure 10: Overall Stability Summary (Resource Comparison)
First 10% Mean
Last 10% Mean
Metric Unit Values
60
50
40
30
20
10
0
23.3
22.6
47.6
49.3
25.4
24.9
60.6
60.5
CPU (%)
RAM (x0.1 %)
GPU (%)
Temp (°C)

(j)

Session Analytics: session_20260601_223652

Long-Duration Parameter Statistics

	Metric	Mean	Median	Min	Max	Std Dev	Variance
1	CPU (%)	21.938	21.600	0.000	44.400	2.433	5.921
2	RAM Used (MB)	15722.671	15763.781	15110.266	16428.199	262.565	68940.297
3	GPU (%)	24.138	24.037	0.000	47.196	3.907	15.267
4	GPU Temp (°C)	60.302	60.278	55.830	66.091	1.039	1.078

(k)

	Resource Metric	First 10% Average	Last 10% Average	Absolute / Pct Change	Status Verdict
1	CPU	23.33	22.57	-3.23%	Stable
2	RAM	15327.88	15899.49	+3.73%	Stable
3	GPU	25.36	24.93	-1.66%	Stable
4	GPU Temp	60.56	60.48	-0.08 °C	Stable

Comparing the initial 10% of the session to the final 10%, the resource metrics showed minimal drift. Memory grew by 3.73%, and GPU temperature shifted by -0.08°C. This demonstrates long-duration equilibrium under load.

(l)

Figure S1. Long-duration operational stability over the 8.38-hour session (22:36–06:59). (a) CPU utilisation (%) over 503 min; mean 21.94%, max 44.40%, std 2.43%. (b) RAM allocation (MB) over time; upward trend slope 94.19 MB/h ($r = 0.868$). (c) GPU compute load (%) over 503 min; mean 24.14%, max 47.20%. (d) GPU core temperature (°C); mean 60.30°C, max 66.09°C, std 1.04°C. (e) VRAM consumption (MB); stable oscillation between 3800–4500 MB. (f) CPU clock frequency (MHz); constant at ~1400 MHz throughout — no throttling. (g) GPU core clock (MHz); sustained ~1800 MHz with no sustained downward drift. (h) RAM linear regression fit (slope 94.19 MB/h); 24-h projection ≈ 2260 MB, within system capacity. (i) GPU temperature vs. clock frequency (throttling check); correlation $r = -0.008$, confirming absence of thermal throttling. (j) First 10% vs. last 10% mean comparison for CPU, RAM, GPU, and temperature; all resources stable. (k) Long-duration parameter statistics: mean, median, min, max, standard deviation, and variance for CPU utilisation, RAM allocation, GPU utilisation, and GPU core temperature over the 8.38-hour session. (l) Long-duration drift analysis: first 10% vs. last 10% session averages for each resource metric, with absolute and percentage change.
